# Supplementary figures and images for: Novel repertoire of tau biosensors to monitor pathological tau transformation and seeding activity in living cells
Source: eLife. 2023 Mar 14;12:e78360. doi: 10.7554/eLife.78360 (PMC10014071; doi:10.7554/eLife.78360)

**Raw unedited gel of Figure 1E**

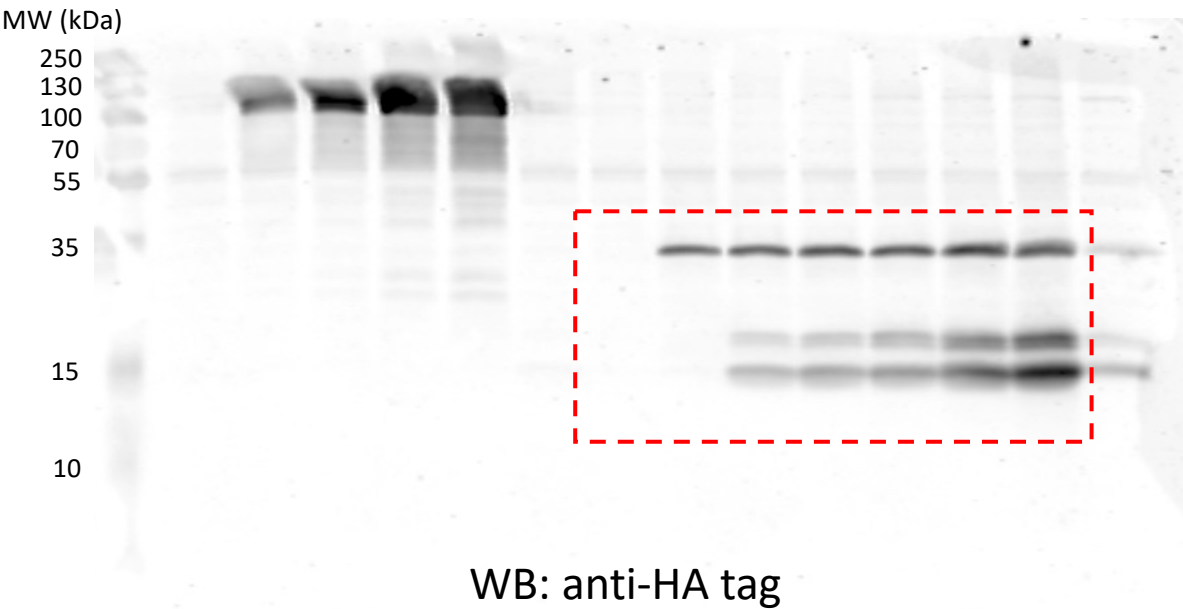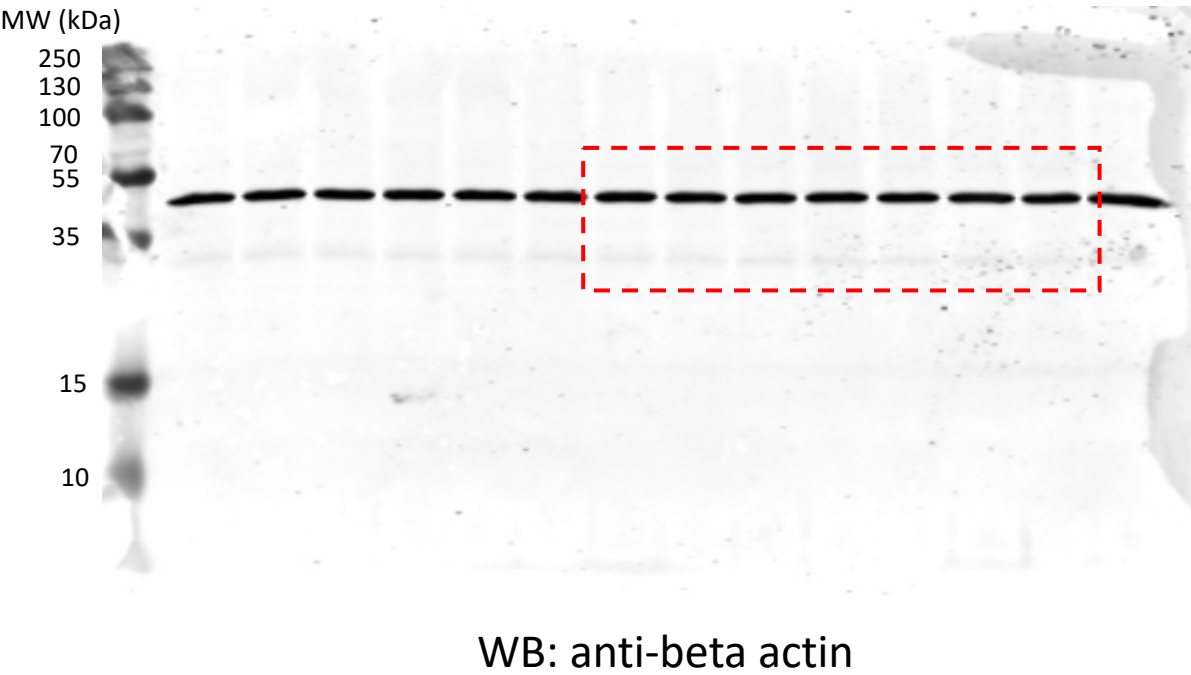

Supplement: Figure 1—source data 1. [file elife-78360-fig1-data1.zip › Fig1-Source_data_1/Fig1E-source_data.pdf]

# Raw unedited gel of Figure 2A

MW (kDa)

250

130

100

70

55

35

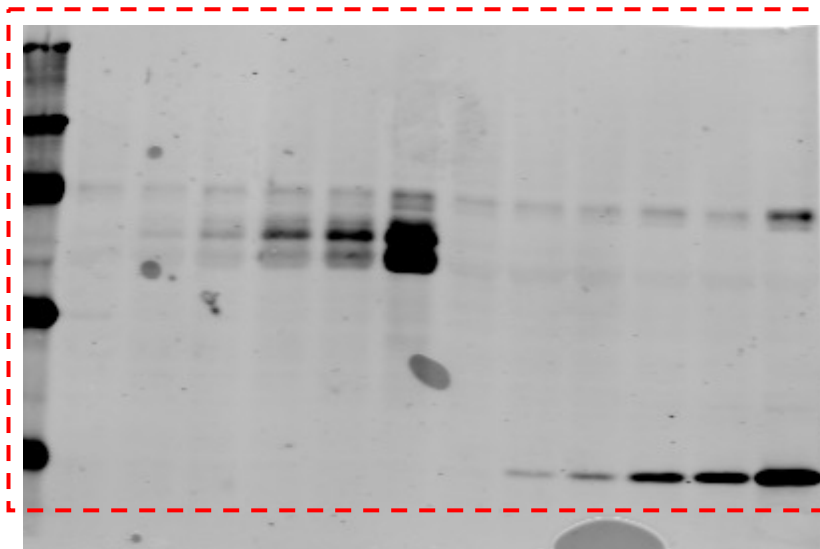

WB: anti-HA tag

Supplement: Figure 2—source data 1. [file elife-78360-fig2-data1.zip › Fig2-Source_data_1/Fig2A-source_data.pdf]

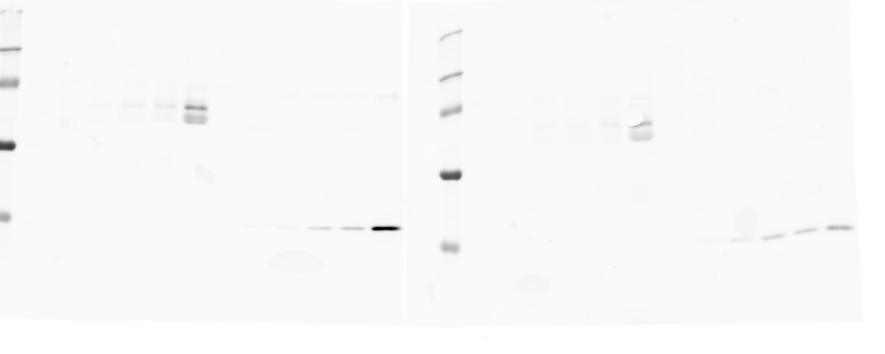

Supplement: Figure 2—source data 1. [file elife-78360-fig2-data1.zip › Fig2-Source_data_1/Fig2A-Copyof700.TIF]

**Raw unedited gel of Figure 2E**

MW (kDa)

250  
130  
100  
70  
55  
35  
15  
10

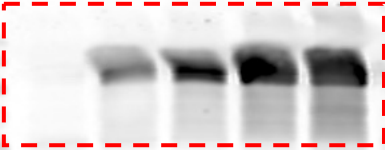

WB: anti-HA tag

MW (kDa)

250  
130  
100  
70  
55  
35  
15  
10

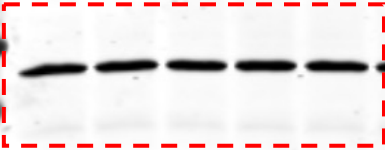

WB: anti-beta actin

Supplement: Figure 2—source data 2. [file elife-78360-fig2-data2.zip › Fig2-Source_data_2/Fig2E-source_data.pdf]

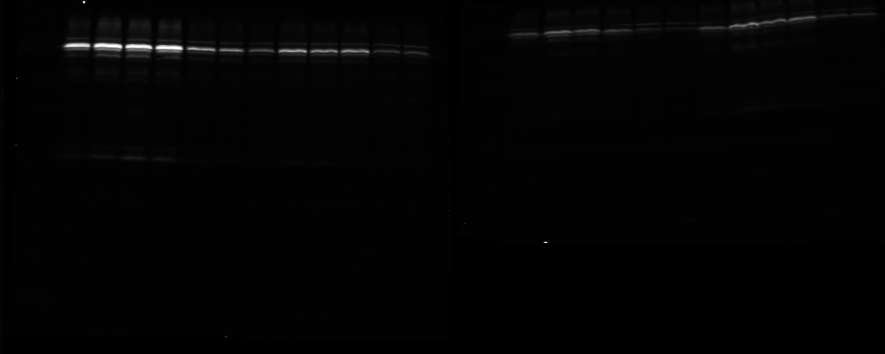

Supplement: Figure 2—source data 3. [file elife-78360-fig2-data3.zip › Fig2-Source_data_3/Fig2K-Copyof800.TIF]

## Raw unedited gel of Figure 2K

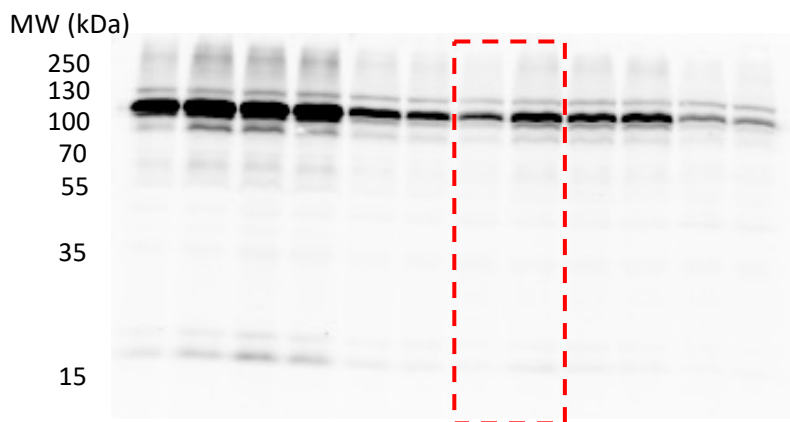

WB: anti-phospho-tau (AT8 antibody)

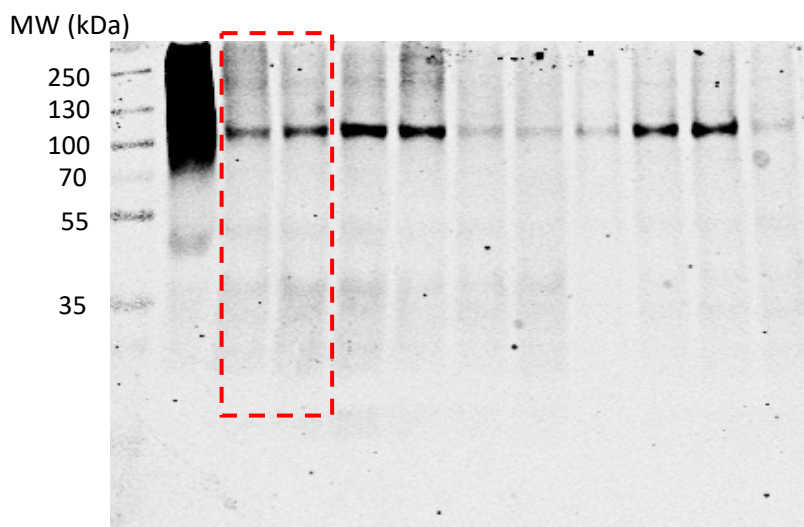

WB: anti-HA tag

Supplement: Figure 2—source data 3. [file elife-78360-fig2-data3.zip › Fig2-Source_data_3/Fig2K_source_data.pdf]

## Raw unedited gel of Figure 2L

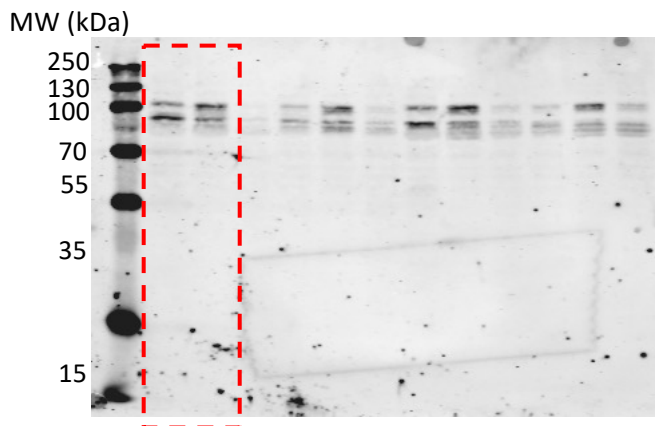

WB: anti-phospho-tau (AT8 antibody)

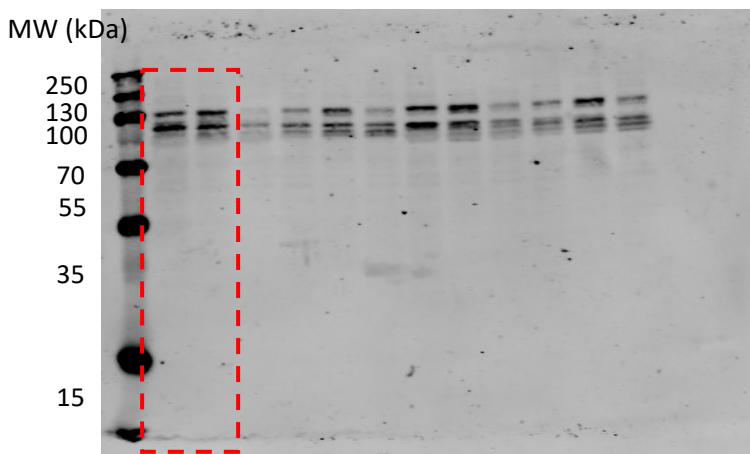

WB: anti-HA tag

Supplement: Figure 2—source data 4. [file elife-78360-fig2-data4.zip › Fig2-Source_data_4/Fig2L-source_data.pdf]

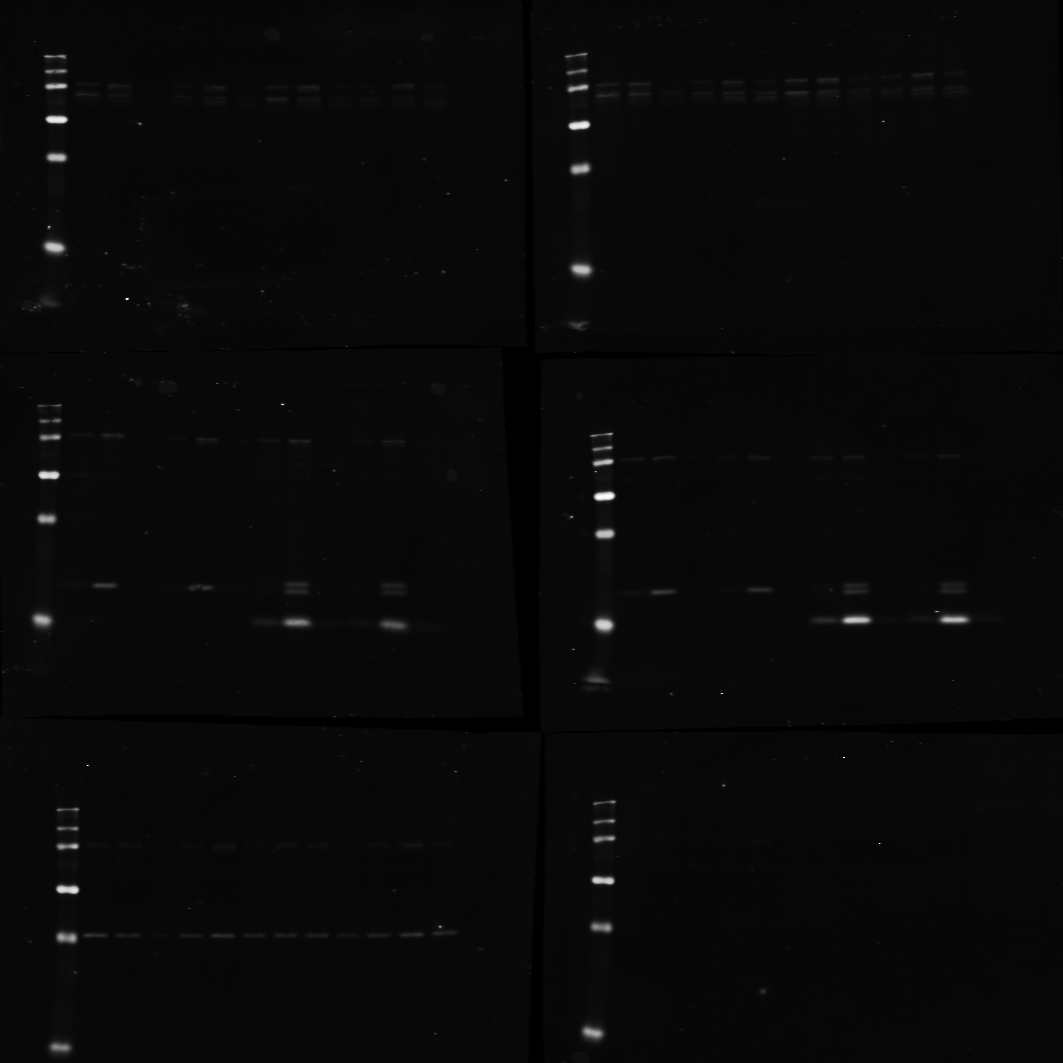

Supplement: Figure 2—source data 4. [file elife-78360-fig2-data4.zip › Fig2-Source_data_4/Fig2L-Copyof700.TIF]

Raw unedited gel of Figure 2M

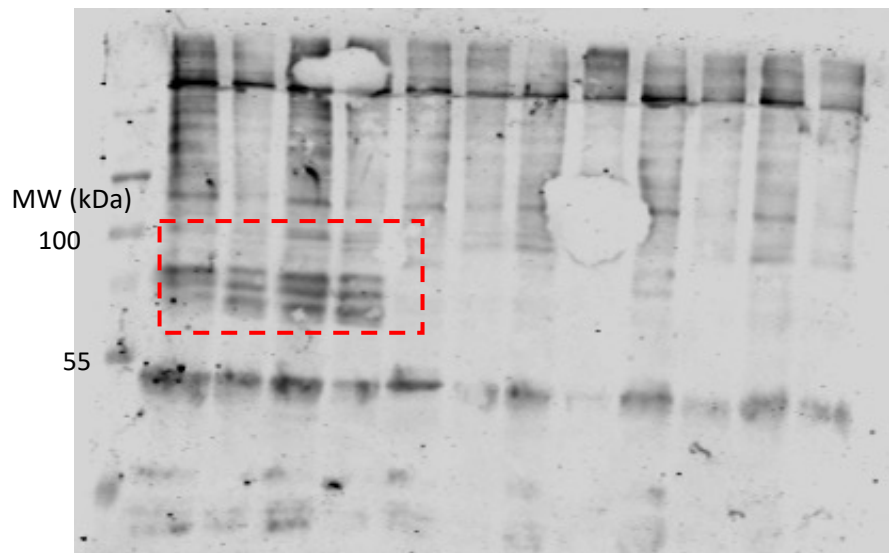

WB: anti-phospho-tau (AT8 antibody)

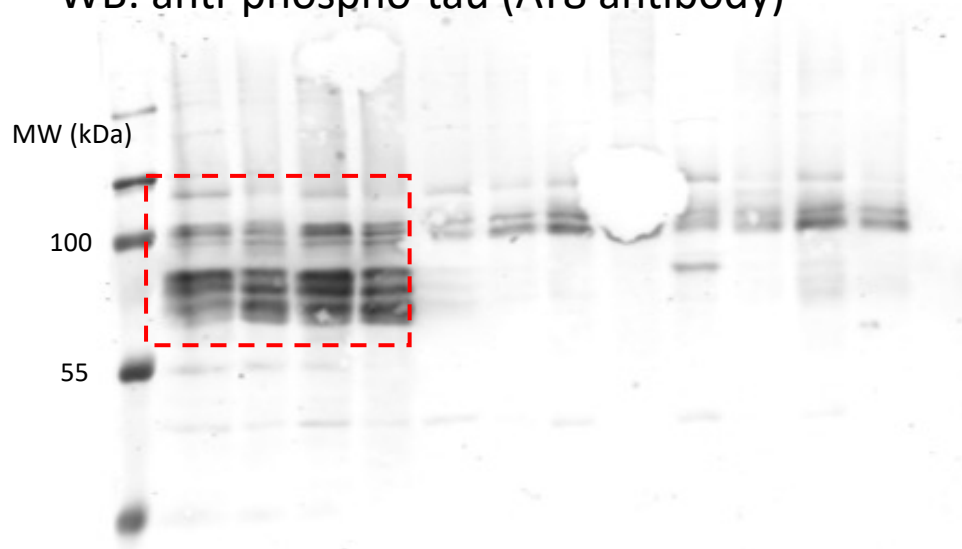

WB: anti-HA tag

Supplement: Figure 2—source data 5. [file elife-78360-fig2-data5.zip › Fig2-Source_data_5/Fig2M-source_data.pdf]

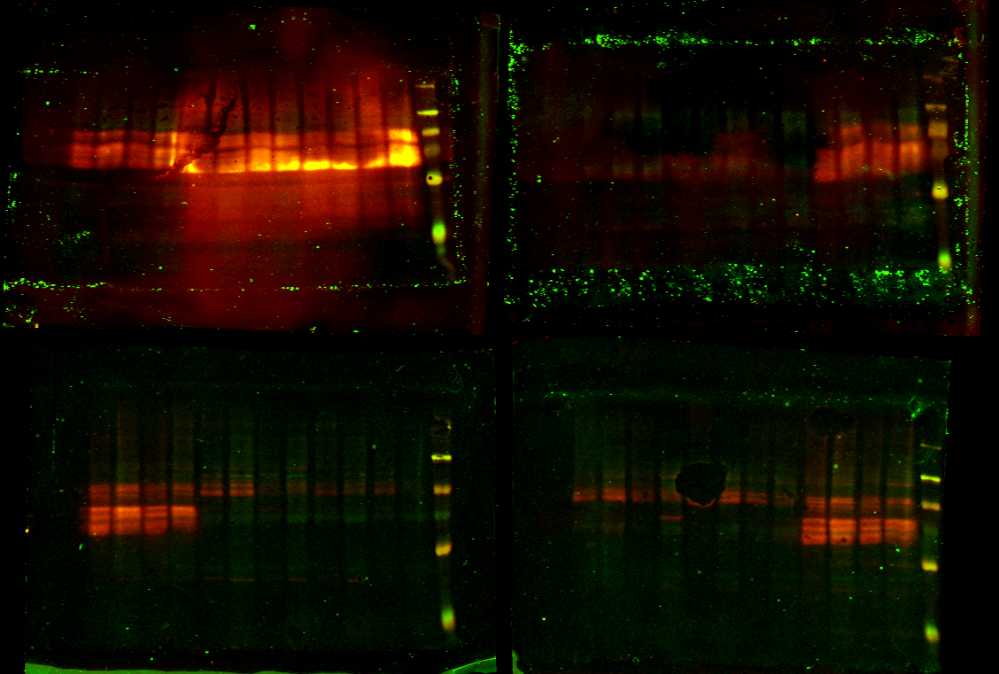

Supplement: Figure 2—source data 5. [file elife-78360-fig2-data5.zip › Fig2-Source_data_5/Fig2M-700-800.png]

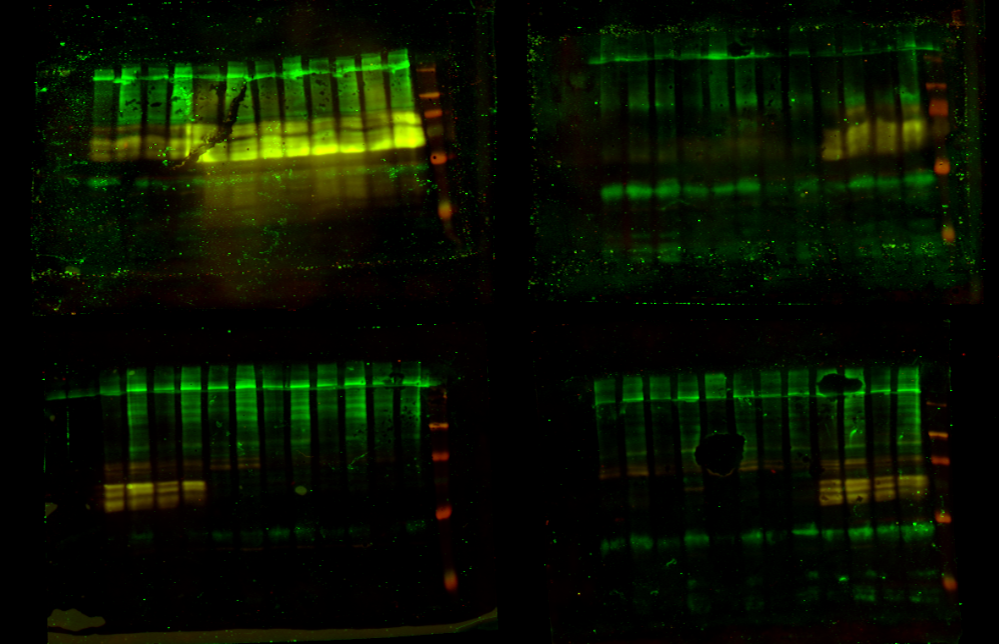

Supplement: Figure 2—source data 5. [file elife-78360-fig2-data5.zip › Fig2-Source_data_5/Fig2M-800.png]

Raw unedited gel of Figure 4A

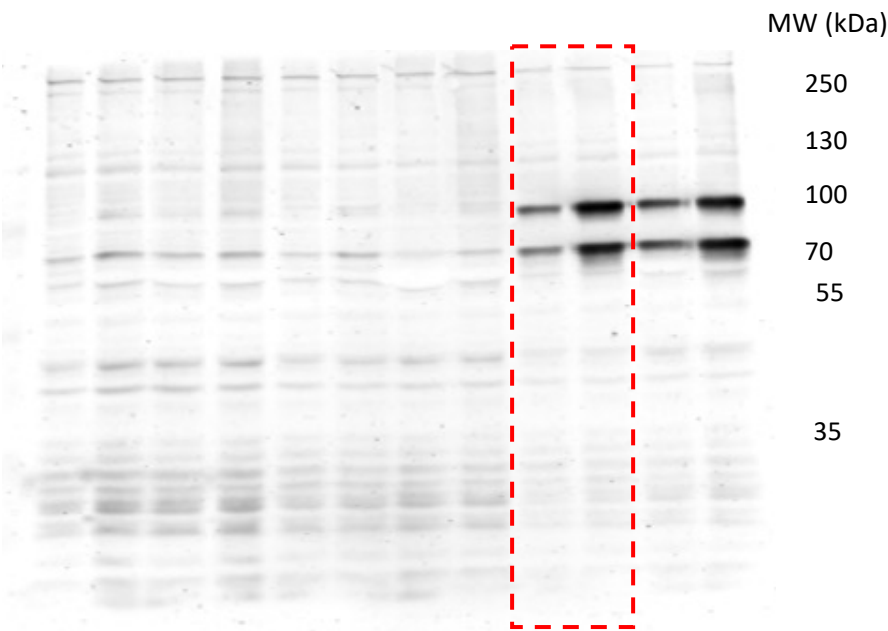

WB: anti-phospho-tau (AT8 antibody)

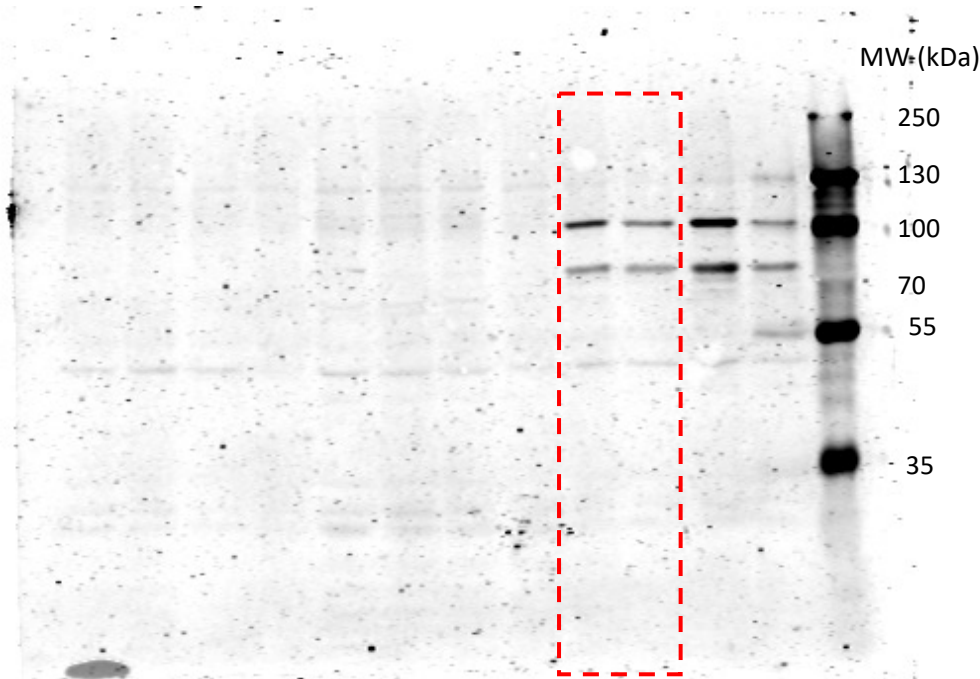

WB: anti-HA tag

Supplement: Figure 4—source data 1. [file elife-78360-fig4-data1.zip › Fig4-Source_data-1/Fig4A-source_data.pdf]

Raw unedited gel of Figure 4B

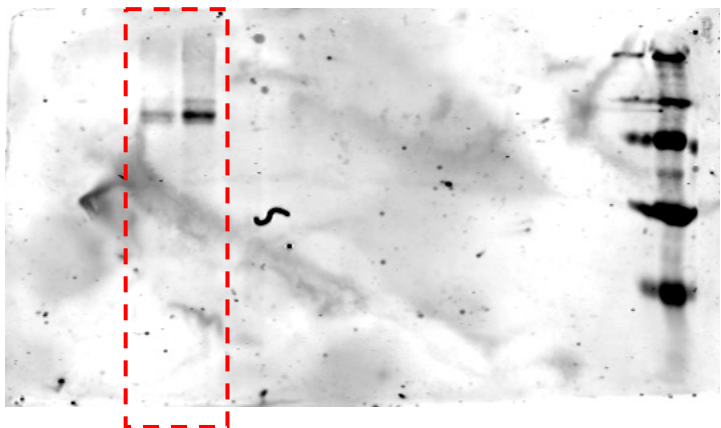

WB: anti-phospho-tau (AT8 antibody)

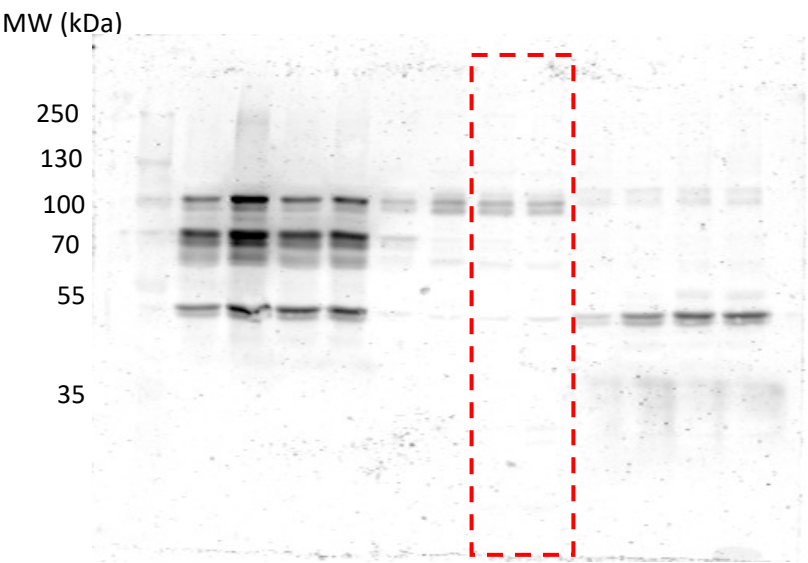

WB: anti-HA tag

Supplement: Figure 4—source data 2. [file elife-78360-fig4-data2.zip › Fig4-Source_data-2/Fig4B-source_data.pdf]
